# Investments

**Published:** 1916-11

**Authors:** 


**A Monthly Review of Medicine and Surgery**

EDITOR AND PUBLISHER, DR. A. L. BENEDICT, 228 Summer St., corner of Elmwood Ave., Buffalo, (Address for all communications. Please make personal and telephone calls before 1 P. M.)

Third, in age, on the western continent. Independent, but supports professional organizations. News limited mainly to Western N. Y. and Penn.

Subscription $2.00 a year; $3.00 for two years in advance. Changes and errors in address or failure or duplication of delivery, should be reported immediately.

Yearly Volume 72 NOVEMBER, 1916 Number 4

### Investments.

You know whether your money comes easy or hard, whether you can afford to gamble with it or whether you want a safe investment. There are some facts so well established that they are almost axioms. 4%, compounded quarterly, can be obtained almost anywhere, with perfect safety, in a bank. A higher rate than 6% is almost always speculative and involves considerable risk. A promotor, that is to say a man who earns (?) his living by speculating with other persons' money, is not absolutely dependable. He cannot be depended upon to spare his personal friends or even relatives. His stock in trade is acquaintance and he cannot be expected to differentiate. The same rule, by the way, applies to the dead beat.

Life insurance appeals especially to those who have dependents, and whose earning power is relatively higher than their capital. It should be considered simply as insurance against want on the part of dependents. Most policies are stated to be good investments in case of survival. Our experience and observation show that they are not. Usually the premiums are returned intact or invested in paid-up insurance or in some other way; probably in the majority of cases, they are returned with a slight increment but seldom or never is the investment value of insurance equal to savings bank interest. Annuity insurance does not surpass the normal interest on an investment on which the capital is not forfeited, until one is well past 50. Money deposited in a good savings bank—and the chances of a bank's being solvent is about the same as for a standard insurance company and better than for one that is not standard—is just as good insurance, as an equal amount payable on death. It is also available in other emergencies than death, which may be a favorable or an unfavorable factor.

The value of stock depends entirely on the willingness of active men in control of a business to work equally for their own interests and those of silent partners who invest no knowledge of the business.. Stock may also impose obligations on the holder. Even when a business is successful, dividends on stock may be suspended for years to enlarge a plant, or excess salaries may be paid to those directly interested or the company may sell out, to the detriment of investors but to the benefit of those conducting the business. Such stocks as have by experience been shown to be good investments are now held at such an advance that the present investor usually gets less interest than on other safe investments.

It is a good rule not to make life insurance payable to a given beneficiary but to one's estate, willing the insurance or any part of it, as desired. For instance, insurance taken for a fiancee or wife may possibly prove to have been taken out for the wrong person and, in general, the death of beneficiary may result in inheritance by persons wholly out of the consideration of the one making the payments. A young man insured his life for the benefit of his mother—a most natural and proper course, apparently. But, by the prior death of his mother without a will, he carried for many years, insurance which was virtually in the interest of two husky brothers-in-law, one of whom would have been honorable enough to transfer it to the proper subsidiary beneficiaries, the other who probably would not have done so.

Government bonds. These pay low interest but are usually exempt from taxation by the corresponding and subordinate government units. They are probably the least troublesome and safest investment for a person with adequate capital but yield only half to two-thirds of what can be derived by ordinary care from other safe investments. Bonds by subordinate branches of the government, as counties, towns and cities, pay slightly higher interest than those issued by a national government and are almost absolutely safe, providing that they have been issued with perfect technical legality. This last proviso, introduces a practical element or risk, not great, but worth careful consideration. The high interest on national war bonds is a recognition of the danger of national defeat and possible insolvency.

Private bonds. These are divided mortgages. There is a technicality which, in some instances, allows what are second liens on property, to be described as first mortgage bonds and this point should be carefully considered. Bonds depend on the property value and solvency of the club, institution or firm issuing them. In the case of any social or philanthropic institution, there is a chance that considerable pressure will be exerted in the future to cancel the indebtedness by surrender of the claim.

Real Estate. This is the soundest of all investments provided that due care is exercised in the purchase. It is a safe rule never to invest in anything at a distance or that has not been personally seen and carefully considered. The man who buys bare land or any other real estate, which he does not intend to manage and use himself, and with the expectation of selling out at a big profit to somebody else, without any exertion on his own part, deserves to lose and usually does. A six per cent. investment, allowing for reinvestment of interest, should double in about 12 years. Undeveloped city lots must double much sooner to allow for ordinary taxes and must double in price immediately, to make up for taxes for paving, sewer and water connections, etc. Hence, the only chance to make easy money on such an investment is to invest at the beginning, at the low price and to sell while the excitement of a boom exists. Do not overrate your judgment in a speculation about which you have no expert knowledge and which is subject to unforeseeable conditions.

As a general rule, it is wise not to buy anything which you cannot use yourself, either personally or as a business. Allowing for taxes, repairs, insurance, vacancy and dead beats, 10% gross rentals, actual and possible of maintenance for the future, are necessary to pay a bare 6% and a great deal of personal attention will be necessary. Look out for "planted" tenants supposed to pay a high rental, for structural defects—even chimney places without chimneys, and a pending order to tear down an old building as unsafe and unsanitary, have been encountered—and for conditions liable to result in future diminution of value. But a home or a desirable building housing several tenants bought on a 10% basis or better, is usually a good investment. Avoid absentee ownership, not for the sake of tenants in this country but for your own sake. Do not buy a farm or a picture show or a factory or anything else adapted to limited use unless you are familiar with the business in question.

Real estate mortgages are, on the whole, the most profitable and most easily managed investments for the man of small capital. Usually, 5% is paid on first mortgages amounting to half of the full nominal value and three-fourths of the sacrifice value. As this interest can be compounded only by reinvestment, it is not so very much better than savings bank interest. 6% can be had on larger first mortgages or on second mortgages. A mortgage should not be placed unless ultimate ownership on foreclosure—involving an additional investment of $150 or more—affords a chance to make good on the 10% basis discussed for actual ownership. In fact, while a mortgage should not be bought with an idea of foreclosure, it should not be placed unless one is willing to take the property if necessary. Do not take a mortgage unless a thorough search as to title has been made and make sure that taxes, and other liens ahead of yours, are promptly paid. While no allowance should be made in taking a mortgage for anything beyond the value of the property itself, the ability and willingness of the owner to pay taxes, interest, etc., is a point to be considered. Many persons ''buy'' a home making a small payment of cash, stand off taxes, interest and repairs as long as possible and then pose as martyrs to the cupidity of a hard-hearted mortgagee, after having had many months' rent at a nominal price. While, of course, first mortgages are generally safer than second, a disproportionate first mortgage running a long term before maturing, so that great depreciation may occur, is often not so safe as a second mortgage which leaves a greater net equity in the property and which is payable in frequent small instalments. And a second mortgage representing part of the obligation in buying a home by an honest and thrifty man, may be much better than a first mortgage which is simply an easy way to raise cash by a man who is extravagant and who has little conception of business obligations.

